# 6β-Acet­oxy-1α,7β,11β,15β-tetra­hydr­oxy-7α,20-ep­oxy-*ent*-kaur-16-ene

**DOI:** 10.1107/S1600536810000231

**Published:** 2010-01-09

**Authors:** Fu-Lin Yan, Peng Li, Xue-Mei Di, Chuang Feng, Rui-Jie Hou

**Affiliations:** a School of Pharmacy, Xinxiang Medical University, Xinxiang, Henan 453003, People’s Republic of China

## Abstract

The title compound, C_22_H_32_O_7_, a natural *ent*-kaurane diterpenoid also referred to as Maoyecrystal F, was obtained from the medicinal plant *Isodon nervosa*. There are four rings with the expected *cis* and *trans* junctions. Cyclohexane ring *A* adopts a chair conformation, rings *B* and *C* adopt boat conformations, while the five-membered ring has an envelope conformation. The mol­ecules stack along the *a* axis in the crystal and are linked together by inter­molecular O—H⋯O hydrogen bonds.

## Related literature

For related literature on the genus *Isodon* and diterpenoids, see: Sun *et al.* (2001 ▶); Zhang *et al.* (2003 ▶); Yan *et al.* (2008 ▶).
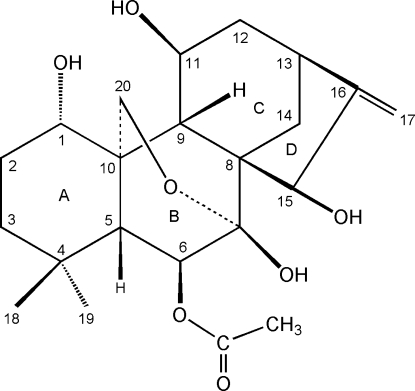


## Experimental

### 

#### Crystal data

C_22_H_32_O_7_
*M* *_r_* = 408.21
Monoclinic, 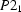

*a* = 9.759 (3) Å
*b* = 6.6712 (17) Å
*c* = 14.927 (4) Å
β = 90.002 (4)°
*V* = 971.8 (4) Å^3^
*Z* = 2
Mo *K*α radiation
μ = 0.10 mm^−1^
*T* = 93 K
0.50 × 0.33 × 0.23 mm

#### Data collection

Rigaku AFC10 Saturn724+ diffractometer
7880 measured reflections
2412 independent reflections
2262 reflections with *I* > 2σ(*I*)
*R*_int_ = 0.025

#### Refinement

*R*[*F* ^2^ > 2σ(*F* ^2^)] = 0.028
*wR*(*F* ^2^) = 0.064
*S* = 1.00
2412 reflections
281 parameters
1 restraint
H atoms treated by a mixture of independent and constrained refinement
Δρ_max_ = 0.25 e Å^−3^
Δρ_min_ = −0.17 e Å^−3^

### 

Data collection: *CrystalClear* (Rigaku, 2008 ▶); cell refinement: *CrystalClear*; data reduction: *CrystalClear*; program(s) used to solve structure: *SHELXS97* (Sheldrick, 2008 ▶); program(s) used to refine structure: *SHELXL97* (Sheldrick, 2008 ▶); molecular graphics: *SHELXTL* (Sheldrick, 2008 ▶); software used to prepare material for publication: *SHELXTL*.

## Supplementary Material

Crystal structure: contains datablocks global, I. DOI: 10.1107/S1600536810000231/hg2621sup1.cif

Structure factors: contains datablocks I. DOI: 10.1107/S1600536810000231/hg2621Isup2.hkl

Additional supplementary materials:  crystallographic information; 3D view; checkCIF report

## Figures and Tables

**Table 1:** Hydrogen-bond geometry (Å, °)

*D*—H⋯*A*	*D*—H	H⋯*A*	*D*⋯*A*	*D*—H⋯*A*
O2—H2*O*⋯O5	0.85 (3)	1.75 (3)	2.5539 (19)	156 (2)
O4—H4*O*⋯O6^i^	0.82 (3)	2.03 (3)	2.843 (2)	172 (2)
O5—H5*O*⋯O2^ii^	0.82 (2)	1.85 (2)	2.6467 (18)	165 (2)
O6—H6*O*⋯O3	0.81 (2)	2.07 (3)	2.7743 (18)	145 (2)

Symmetry codes: (i) 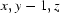; (ii) 
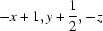
.

**Fig. 1.**
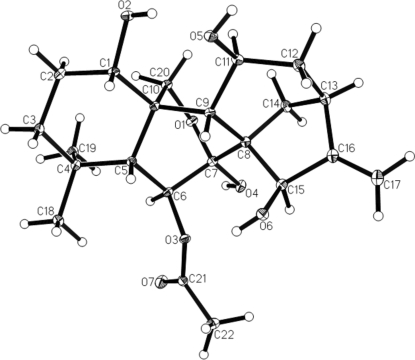
A view of the molecular structure of compound. Displacement ellipsoids are drawn at the 50% probability level.

**Fig. 2.**
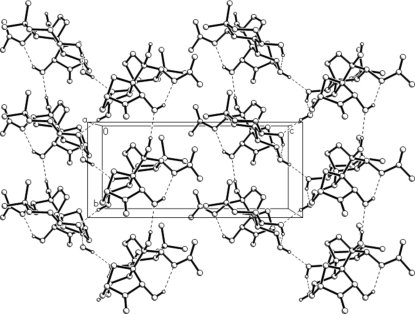
The crystal packing of compound, viewed along the a axis, showing the O—H···O hydrogen bonds as dashed lines.

## supplementary crystallographic information

### Comment

The title compound, 6β-Acetoxy-1α,7β,11β,15β-tetrahydroxy- 7α,20-epoxy-*ent*-kaur-16-ene, is a natural *ent*-kaurane diterpenoid isolated from the medicinal plant *Isodon nervosa*. It is widely distributed in China, and has long been used as a Chinese folk medicine in the treatment of acute jaundice, hepatitis and acute cholecystitis. We re-examined the leaves of *Isodon nervosus* collected in Henan province of China and obtained the *ent*-kaurane diterpenoid, named Maoyecrystal *F* (Yan *et al.*, 2008). The compound has also been isolated from *Isodon japonica* and its structure was postulated from spectroscopic methods (Zhang *et al.*, 2003). Its X-ray crystallographic analysis confirms this proposed molecular structure (Fig. 1). In the structure there is a *trans* junction between ring *A* (C1—C5/C10) and ring *B* (C5—C10); *cis* junctions are present between ring *B* and ring *C* (C8/C9/C11—C14), and ring *C* and ring *D* (C8/C13—C16). Ring *A* adopts chair conformation, with an average torsion angles of 53.99 (19) °. Rings *B* and *C* adopt boat conformation because of the formation of the oxygen bridge at C-7 and C-20. Ring *D* shows an evenlope conformation. In addition, the six-membered rings O1/C20/C10/C5—C7 and O1/C7—C10/C20 both adopt boat conformations. The four hydroxy groups at C1, C7, C11 and C15 adopt α, β, β, β-orientations respectively, an acetoxy group at C6 adopt β-orientation. Bond lengths and angles are within expected aranges (Allen *et al.*, 1987), with averages values (Å): C*sp*^3^—C*sp*^3^ = 1.544 (2), C*sp*^3^—C*sp*^2^ = 1.516 (2), C*sp*^2^—C*sp*^2^ (CC) = 1.323 (3), C*sp*^3^—O = 1.434 (2).

The compound contains ten chiral centers at C1(*S*), C5(*R*), C6(*S*), C7(*S*), C8(*S*), C9(*S*), C10(*S*), C11(*S*),C13(*S*) and C15(*R*). Although the absolute configuration could not be reliably determined from anomalous dispersion effects, the negative optical rotation showed this compound to be in the *ent*-kaurane series as reported in genus *Isodon* (Sun *et al.*, 2001), rather than in the kaurane series, allowing us to assign the correct configuration. In the crystal structure, intermolecular O—H···O hydrogen bond (table 1) are effective in the stabilization of the structure and are responsible for the formation of a three-dimensional network (Fig. 2).

### Experimental

The dried and crushed leaves of *Isodon nervosa* (12 kg, collected from Henan Province, China) were extracted three times with Me_2_CO/H_2_O (7:3, *v*/*v*) at room temperature over a period of six days. The extract was filtered and the solvent was removed under reduced pressure. The residue was then partitioned between water and AcOEt. After removal of the solvent, the AcOEt residue was separated by repeated silica gel (200–300 mesh) column chromatography and recrystallization from CHCl_3_/CH_3_OH(8:1), giving 35 mg of the title compound (m.p. 489–491 K. Optical rotation: [α]_D_^20^ -6.1 ° (c 0.52, CH_3_OH). Crystals suitable for X-ray analysis were obtained by slow evaporation of a solution of the title compound in CH_3_OH at room temperature.

### Refinement

All H atoms were included in calculated positions and refined as riding atoms, with C—H = 0.98Å (CH_3_), 0.99Å (CH_2_), 0.95Å (═CH_2_), 1.00Å (CH), and O—H = 0.82Å, and with *U*_iso_(H) = 1.2 *U*_eq_(C). In the absence of significant anomalous scattering effects, Friedel pairs were merged. The choice of enantiomer was based on comparison of the optical rotation with that of related compounds having known stereochemistry.

### Crystal data

**Table:**

C_22_H_32_O_7_	*F*(000) = 440
*M**_r_* = 408.21	*D*_x_ = 1.396 Mg m^−^^3^
Monoclinic, *P*2_1_	Melting point = 489–491 K
Hall symbol: P 2yb	Mo *K*α radiation, λ = 0.71073 Å
*a* = 9.759 (3) Å	Cell parameters from 3508 reflections
*b* = 6.6712 (17) Å	θ = 3.3–27.5°
*c* = 14.927 (4) Å	µ = 0.10 mm^−^^1^
β = 90.002 (4)°	*T* = 93 K
*V* = 971.8 (4) Å^3^	Prism, colorless
*Z* = 2	0.50 × 0.33 × 0.23 mm

### Data collection

**Table:**

Rigaku AFC10 Saturn724+ diffractometer	2262 reflections with *I* > 2σ(*I*)
Radiation source: rotating anode	*R*_int_ = 0.025
graphite	θ_max_ = 27.5°, θ_min_ = 3.3°
Detector resolution: 28.5714 pixels mm^-1^	*h* = −9→12
ω scans	*k* = −8→8
7880 measured reflections	*l* = −19→19
2412 independent reflections	

### Refinement

**Table:**

Refinement on *F*^2^	Primary atom site location: structure-invariant direct methods
Least-squares matrix: full	Secondary atom site location: difference Fourier map
*R*[*F*^2^ > 2σ(*F*^2^)] = 0.028	Hydrogen site location: inferred from neighbouring sites
*wR*(*F*^2^) = 0.064	H atoms treated by a mixture of independent and constrained refinement
*S* = 1.00	*w* = 1/[σ^2^(*F*_o_^2^) + (0.0336*P*)^2^ + 0.163*P*] where *P* = (*F*_o_^2^ + 2*F*_c_^2^)/3
2412 reflections	(Δ/σ)_max_ < 0.001
281 parameters	Δρ_max_ = 0.25 e Å^−^^3^
1 restraint	Δρ_min_ = −0.17 e Å^−^^3^

### Special details

**Table:**

Geometry. All e.s.d.'s (except the e.s.d. in the dihedral angle between two l.s. planes) are estimated using the full covariance matrix. The cell e.s.d.'s are taken into account individually in the estimation of e.s.d.'s in distances, angles and torsion angles; correlations between e.s.d.'s in cell parameters are only used when they are defined by crystal symmetry. An approximate (isotropic) treatment of cell e.s.d.'s is used for estimating e.s.d.'s involving l.s. planes.
Refinement. Refinement of *F*^2^ against ALL reflections. The weighted *R*-factor *wR* and goodness of fit *S* are based on *F*^2^, conventional *R*-factors *R* are based on *F*, with *F* set to zero for negative *F*^2^. The threshold expression of *F*^2^ >σ(*F*^2^) is used only for calculating *R*-factors(gt) *etc*. and is not relevant to the choice of reflections for refinement. *R*-factors based on *F*^2^ are statistically about twice as large as those based on *F*, and *R*-factors based on ALL data will be even larger.

### Fractional atomic coordinates and isotropic or equivalent isotropic displacement parameters (Å^2^)

**Table:**

	*x*	*y*	*z*	*U*_iso_*/*U*_eq_	
O1	0.71560 (11)	0.24172 (19)	0.19745 (8)	0.0137 (3)	
O2	0.39948 (13)	0.6243 (2)	0.07687 (8)	0.0153 (3)	
O3	0.76328 (11)	0.54105 (18)	0.39884 (7)	0.0130 (3)	
O4	0.91072 (12)	0.2840 (2)	0.27898 (9)	0.0146 (3)	
O5	0.57949 (12)	0.90169 (19)	0.06925 (8)	0.0149 (3)	
O6	0.87086 (12)	0.86646 (19)	0.30694 (8)	0.0143 (3)	
O7	0.78961 (13)	0.2859 (2)	0.49762 (9)	0.0232 (3)	
C1	0.41761 (16)	0.6066 (3)	0.17186 (11)	0.0124 (3)	
H1	0.4059	0.7432	0.1983	0.015*	
C2	0.30078 (17)	0.4772 (3)	0.20568 (11)	0.0157 (4)	
H2A	0.3104	0.3398	0.1814	0.019*	
H2B	0.2125	0.5326	0.1843	0.019*	
C3	0.30058 (17)	0.4693 (3)	0.30758 (11)	0.0162 (4)	
H3A	0.2223	0.3867	0.3280	0.019*	
H3B	0.2878	0.6065	0.3314	0.019*	
C4	0.43380 (16)	0.3814 (3)	0.34543 (11)	0.0132 (3)	
C5	0.55753 (16)	0.4991 (3)	0.30404 (11)	0.0116 (3)	
H5	0.5535	0.6368	0.3304	0.014*	
C6	0.69539 (16)	0.4098 (3)	0.33418 (10)	0.0119 (3)	
H6	0.6780	0.2771	0.3632	0.014*	
C7	0.78918 (16)	0.3783 (3)	0.25387 (11)	0.0121 (3)	
C8	0.82107 (16)	0.5716 (3)	0.20203 (11)	0.0116 (3)	
C9	0.68182 (16)	0.6785 (3)	0.17895 (11)	0.0111 (3)	
H9	0.6720	0.7965	0.2198	0.013*	
C10	0.56065 (16)	0.5289 (3)	0.19985 (11)	0.0111 (3)	
C11	0.68778 (17)	0.7582 (3)	0.08150 (11)	0.0132 (3)	
H11	0.6716	0.6433	0.0398	0.016*	
C12	0.82467 (17)	0.8558 (3)	0.05703 (11)	0.0166 (4)	
H12A	0.8296	0.9898	0.0854	0.020*	
H12B	0.8285	0.8750	−0.0087	0.020*	
C13	0.95062 (17)	0.7303 (3)	0.08700 (11)	0.0160 (4)	
H13	1.0228	0.7243	0.0395	0.019*	
C14	0.90325 (17)	0.5203 (3)	0.11600 (11)	0.0143 (3)	
H14A	0.9820	0.4315	0.1292	0.017*	
H14B	0.8444	0.4569	0.0700	0.017*	
C15	0.92640 (16)	0.7137 (3)	0.24989 (11)	0.0132 (3)	
H15	0.9913	0.6298	0.2857	0.016*	
C16	1.00555 (17)	0.8127 (3)	0.17408 (11)	0.0152 (4)	
C17	1.10478 (18)	0.9460 (3)	0.18423 (13)	0.0210 (4)	
H17A	1.1495	1.0001	0.1332	0.025*	
H17B	1.1313	0.9875	0.2426	0.025*	
C18	0.43741 (17)	0.4168 (3)	0.44718 (11)	0.0165 (4)	
H18A	0.3619	0.3437	0.4756	0.020*	
H18B	0.4278	0.5604	0.4595	0.020*	
H18C	0.5248	0.3689	0.4713	0.020*	
C19	0.43608 (18)	0.1517 (3)	0.32907 (12)	0.0169 (4)	
H19A	0.3618	0.0884	0.3630	0.020*	
H19B	0.5243	0.0970	0.3488	0.020*	
H19C	0.4236	0.1247	0.2651	0.020*	
C20	0.59817 (16)	0.3306 (3)	0.15381 (11)	0.0124 (3)	
H20A	0.5194	0.2372	0.1568	0.015*	
H20B	0.6194	0.3554	0.0899	0.015*	
C21	0.81536 (17)	0.4540 (3)	0.47425 (11)	0.0158 (4)	
C22	0.90964 (19)	0.5955 (3)	0.52190 (12)	0.0221 (4)	
H22A	0.9963	0.6051	0.4890	0.027*	
H22B	0.9273	0.5457	0.5826	0.027*	
H22C	0.8670	0.7283	0.5253	0.027*	
H5O	0.597 (2)	0.957 (4)	0.0218 (15)	0.022 (6)*	
H6O	0.834 (2)	0.812 (4)	0.3491 (15)	0.025 (6)*	
H2O	0.444 (2)	0.730 (4)	0.0632 (15)	0.029 (6)*	
H4O	0.892 (2)	0.166 (4)	0.2884 (16)	0.031 (7)*	

### Atomic displacement parameters (Å^2^)

**Table:**

	*U*^11^	*U*^22^	*U*^33^	*U*^12^	*U*^13^	*U*^23^
O1	0.0143 (5)	0.0100 (6)	0.0166 (6)	0.0011 (5)	−0.0030 (4)	−0.0022 (5)
O2	0.0183 (6)	0.0157 (7)	0.0119 (6)	−0.0004 (5)	−0.0039 (5)	−0.0004 (5)
O3	0.0139 (5)	0.0136 (6)	0.0116 (5)	−0.0020 (5)	−0.0024 (4)	−0.0010 (5)
O4	0.0116 (5)	0.0100 (7)	0.0223 (6)	0.0012 (5)	−0.0015 (5)	0.0024 (5)
O5	0.0174 (6)	0.0129 (6)	0.0143 (6)	0.0027 (5)	−0.0016 (5)	0.0034 (5)
O6	0.0178 (6)	0.0108 (6)	0.0145 (6)	−0.0008 (5)	0.0011 (5)	−0.0015 (5)
O7	0.0194 (6)	0.0275 (8)	0.0227 (7)	−0.0025 (6)	−0.0052 (5)	0.0115 (6)
C1	0.0137 (8)	0.0126 (8)	0.0109 (7)	0.0022 (7)	−0.0028 (6)	−0.0008 (6)
C2	0.0127 (7)	0.0170 (9)	0.0174 (8)	−0.0002 (7)	−0.0035 (6)	−0.0001 (7)
C3	0.0116 (7)	0.0191 (9)	0.0179 (8)	0.0000 (7)	0.0011 (6)	0.0005 (7)
C4	0.0118 (7)	0.0144 (9)	0.0135 (7)	−0.0011 (7)	−0.0003 (6)	−0.0003 (7)
C5	0.0118 (7)	0.0112 (8)	0.0117 (7)	0.0005 (7)	−0.0011 (6)	−0.0012 (6)
C6	0.0116 (7)	0.0111 (8)	0.0130 (7)	−0.0015 (7)	−0.0026 (6)	0.0002 (7)
C7	0.0122 (7)	0.0090 (8)	0.0150 (7)	0.0012 (7)	−0.0027 (6)	−0.0013 (7)
C8	0.0116 (7)	0.0100 (8)	0.0132 (7)	0.0013 (6)	0.0007 (6)	−0.0001 (7)
C9	0.0122 (7)	0.0095 (8)	0.0115 (7)	0.0013 (6)	−0.0013 (6)	−0.0003 (6)
C10	0.0114 (7)	0.0102 (8)	0.0116 (7)	0.0003 (7)	−0.0011 (6)	0.0001 (6)
C11	0.0160 (7)	0.0117 (8)	0.0119 (7)	0.0033 (7)	0.0000 (6)	−0.0002 (6)
C12	0.0179 (8)	0.0157 (9)	0.0161 (8)	−0.0009 (8)	0.0016 (6)	0.0014 (7)
C13	0.0157 (8)	0.0155 (9)	0.0169 (8)	0.0006 (7)	0.0042 (6)	0.0010 (7)
C14	0.0149 (7)	0.0128 (9)	0.0154 (8)	0.0023 (7)	0.0031 (6)	−0.0019 (7)
C15	0.0127 (7)	0.0112 (9)	0.0158 (8)	0.0018 (7)	−0.0011 (6)	0.0000 (7)
C16	0.0133 (7)	0.0123 (9)	0.0199 (8)	0.0038 (7)	0.0017 (6)	0.0007 (7)
C17	0.0184 (8)	0.0189 (10)	0.0257 (9)	−0.0017 (8)	0.0036 (7)	0.0006 (8)
C18	0.0158 (8)	0.0189 (10)	0.0147 (8)	−0.0005 (7)	0.0017 (6)	0.0006 (7)
C19	0.0167 (8)	0.0156 (9)	0.0186 (8)	−0.0040 (7)	−0.0005 (7)	0.0028 (7)
C20	0.0134 (7)	0.0103 (8)	0.0136 (7)	0.0010 (7)	−0.0014 (6)	−0.0017 (7)
C21	0.0115 (7)	0.0236 (10)	0.0124 (7)	0.0018 (7)	−0.0002 (6)	0.0026 (7)
C22	0.0190 (9)	0.0298 (11)	0.0175 (8)	0.0000 (8)	−0.0049 (7)	−0.0014 (8)

### Geometric parameters (Å, °)

**Table:**

O1—C7	1.434 (2)	C8—C9	1.573 (2)
O1—C20	1.4453 (19)	C9—C11	1.550 (2)
O2—C1	1.4337 (19)	C9—C10	1.578 (2)
O2—H2O	0.85 (3)	C9—H9	1.0000
O3—C21	1.365 (2)	C10—C20	1.535 (2)
O3—C6	1.4620 (19)	C11—C12	1.530 (2)
O4—C7	1.394 (2)	C11—H11	1.0000
O4—H4O	0.82 (3)	C12—C13	1.553 (2)
O5—C11	1.438 (2)	C12—H12A	0.9900
O5—H5O	0.82 (2)	C12—H12B	0.9900
O6—C15	1.434 (2)	C13—C16	1.510 (2)
O6—H6O	0.81 (2)	C13—C14	1.537 (3)
O7—C21	1.201 (2)	C13—H13	1.0000
C1—C2	1.517 (2)	C14—H14A	0.9900
C1—C10	1.546 (2)	C14—H14B	0.9900
C1—H1	1.0000	C15—C16	1.521 (2)
C2—C3	1.522 (2)	C15—H15	1.0000
C2—H2A	0.9900	C16—C17	1.323 (3)
C2—H2B	0.9900	C17—H17A	0.9500
C3—C4	1.534 (2)	C17—H17B	0.9500
C3—H3A	0.9900	C18—H18A	0.9800
C3—H3B	0.9900	C18—H18B	0.9800
C4—C18	1.538 (2)	C18—H18C	0.9800
C4—C19	1.552 (3)	C19—H19A	0.9800
C4—C5	1.567 (2)	C19—H19B	0.9800
C5—C6	1.539 (2)	C19—H19C	0.9800
C5—C10	1.568 (2)	C20—H20A	0.9900
C5—H5	1.0000	C20—H20B	0.9900
C6—C7	1.523 (2)	C21—C22	1.498 (3)
C6—H6	1.0000	C22—H22A	0.9800
C7—C8	1.535 (2)	C22—H22B	0.9800
C8—C14	1.552 (2)	C22—H22C	0.9800
C8—C15	1.570 (2)		
			
C7—O1—C20	113.65 (13)	C5—C10—C9	106.89 (12)
C1—O2—H2O	104.1 (16)	O5—C11—C12	109.13 (14)
C21—O3—C6	117.28 (14)	O5—C11—C9	108.68 (13)
C7—O4—H4O	106.7 (17)	C12—C11—C9	113.75 (13)
C11—O5—H5O	104.9 (16)	O5—C11—H11	108.4
C15—O6—H6O	108.2 (17)	C12—C11—H11	108.4
O2—C1—C2	106.46 (13)	C9—C11—H11	108.4
O2—C1—C10	113.96 (13)	C11—C12—C13	113.12 (14)
C2—C1—C10	113.43 (14)	C11—C12—H12A	109.0
O2—C1—H1	107.6	C13—C12—H12A	109.0
C2—C1—H1	107.6	C11—C12—H12B	109.0
C10—C1—H1	107.6	C13—C12—H12B	109.0
C1—C2—C3	110.70 (14)	H12A—C12—H12B	107.8
C1—C2—H2A	109.5	C16—C13—C14	101.32 (14)
C3—C2—H2A	109.5	C16—C13—C12	109.43 (15)
C1—C2—H2B	109.5	C14—C13—C12	109.54 (14)
C3—C2—H2B	109.5	C16—C13—H13	112.0
H2A—C2—H2B	108.1	C14—C13—H13	112.0
C2—C3—C4	112.34 (14)	C12—C13—H13	112.0
C2—C3—H3A	109.1	C13—C14—C8	100.80 (13)
C4—C3—H3A	109.1	C13—C14—H14A	111.6
C2—C3—H3B	109.1	C8—C14—H14A	111.6
C4—C3—H3B	109.1	C13—C14—H14B	111.6
H3A—C3—H3B	107.9	C8—C14—H14B	111.6
C3—C4—C18	108.92 (14)	H14A—C14—H14B	109.4
C3—C4—C19	109.37 (15)	O6—C15—C16	108.98 (15)
C18—C4—C19	107.88 (15)	O6—C15—C8	116.86 (13)
C3—C4—C5	108.42 (14)	C16—C15—C8	104.85 (13)
C18—C4—C5	107.14 (13)	O6—C15—H15	108.6
C19—C4—C5	114.95 (14)	C16—C15—H15	108.6
C6—C5—C4	111.35 (14)	C8—C15—H15	108.6
C6—C5—C10	108.79 (13)	C17—C16—C13	127.09 (17)
C4—C5—C10	117.99 (13)	C17—C16—C15	125.33 (16)
C6—C5—H5	106.0	C13—C16—C15	107.58 (14)
C4—C5—H5	106.0	C16—C17—H17A	120.0
C10—C5—H5	106.0	C16—C17—H17B	120.0
O3—C6—C7	109.26 (12)	H17A—C17—H17B	120.0
O3—C6—C5	110.92 (14)	C4—C18—H18A	109.5
C7—C6—C5	110.41 (13)	C4—C18—H18B	109.5
O3—C6—H6	108.7	H18A—C18—H18B	109.5
C7—C6—H6	108.7	C4—C18—H18C	109.5
C5—C6—H6	108.7	H18A—C18—H18C	109.5
O4—C7—O1	107.27 (13)	H18B—C18—H18C	109.5
O4—C7—C6	111.22 (13)	C4—C19—H19A	109.5
O1—C7—C6	104.41 (12)	C4—C19—H19B	109.5
O4—C7—C8	110.02 (13)	H19A—C19—H19B	109.5
O1—C7—C8	109.84 (13)	C4—C19—H19C	109.5
C6—C7—C8	113.76 (14)	H19A—C19—H19C	109.5
C7—C8—C14	109.67 (14)	H19B—C19—H19C	109.5
C7—C8—C15	114.24 (13)	O1—C20—C10	109.92 (12)
C14—C8—C15	99.86 (13)	O1—C20—H20A	109.7
C7—C8—C9	108.42 (13)	C10—C20—H20A	109.7
C14—C8—C9	111.41 (13)	O1—C20—H20B	109.7
C15—C8—C9	113.04 (14)	C10—C20—H20B	109.7
C11—C9—C8	109.19 (12)	H20A—C20—H20B	108.2
C11—C9—C10	115.50 (13)	O7—C21—O3	123.98 (17)
C8—C9—C10	108.50 (13)	O7—C21—C22	125.39 (16)
C11—C9—H9	107.8	O3—C21—C22	110.63 (16)
C8—C9—H9	107.8	C21—C22—H22A	109.5
C10—C9—H9	107.8	C21—C22—H22B	109.5
C20—C10—C1	112.54 (13)	H22A—C22—H22B	109.5
C20—C10—C5	109.85 (14)	C21—C22—H22C	109.5
C1—C10—C5	107.03 (12)	H22A—C22—H22C	109.5
C20—C10—C9	106.13 (13)	H22B—C22—H22C	109.5
C1—C10—C9	114.25 (14)		
			
O2—C1—C2—C3	−173.23 (14)	C2—C1—C10—C9	−170.68 (14)
C10—C1—C2—C3	60.63 (19)	C6—C5—C10—C20	54.20 (17)
C1—C2—C3—C4	−60.1 (2)	C4—C5—C10—C20	−73.84 (17)
C2—C3—C4—C18	168.91 (15)	C6—C5—C10—C1	176.66 (14)
C2—C3—C4—C19	−73.40 (19)	C4—C5—C10—C1	48.62 (19)
C2—C3—C4—C5	52.65 (19)	C6—C5—C10—C9	−60.52 (17)
C3—C4—C5—C6	−176.20 (14)	C4—C5—C10—C9	171.44 (14)
C18—C4—C5—C6	66.39 (18)	C11—C9—C10—C20	73.03 (17)
C19—C4—C5—C6	−53.47 (19)	C8—C9—C10—C20	−49.90 (16)
C3—C4—C5—C10	−49.38 (19)	C11—C9—C10—C1	−51.57 (18)
C18—C4—C5—C10	−166.79 (15)	C8—C9—C10—C1	−174.50 (13)
C19—C4—C5—C10	73.35 (19)	C11—C9—C10—C5	−169.77 (14)
C21—O3—C6—C7	−104.90 (15)	C8—C9—C10—C5	67.31 (16)
C21—O3—C6—C5	133.16 (14)	C8—C9—C11—O5	−163.90 (13)
C4—C5—C6—O3	−107.33 (15)	C10—C9—C11—O5	73.54 (17)
C10—C5—C6—O3	120.97 (14)	C8—C9—C11—C12	−42.11 (19)
C4—C5—C6—C7	131.41 (15)	C10—C9—C11—C12	−164.67 (15)
C10—C5—C6—C7	−0.29 (19)	O5—C11—C12—C13	167.63 (13)
C20—O1—C7—O4	−170.16 (13)	C9—C11—C12—C13	46.1 (2)
C20—O1—C7—C6	71.74 (15)	C11—C12—C13—C16	−98.54 (17)
C20—O1—C7—C8	−50.61 (16)	C11—C12—C13—C14	11.7 (2)
O3—C6—C7—O4	62.10 (17)	C16—C13—C14—C8	47.88 (15)
C5—C6—C7—O4	−175.65 (14)	C12—C13—C14—C8	−67.65 (16)
O3—C6—C7—O1	177.48 (13)	C7—C8—C14—C13	−168.02 (13)
C5—C6—C7—O1	−60.27 (17)	C15—C8—C14—C13	−47.72 (15)
O3—C6—C7—C8	−62.79 (17)	C9—C8—C14—C13	71.94 (16)
C5—C6—C7—C8	59.46 (18)	C7—C8—C15—O6	−92.48 (18)
O4—C7—C8—C14	60.79 (17)	C14—C8—C15—O6	150.59 (14)
O1—C7—C8—C14	−57.07 (17)	C9—C8—C15—O6	32.1 (2)
C6—C7—C8—C14	−173.68 (13)	C7—C8—C15—C16	146.76 (14)
O4—C7—C8—C15	−50.34 (18)	C14—C8—C15—C16	29.83 (16)
O1—C7—C8—C15	−168.19 (13)	C9—C8—C15—C16	−88.63 (15)
C6—C7—C8—C15	75.20 (17)	C14—C13—C16—C17	150.63 (18)
O4—C7—C8—C9	−177.37 (13)	C12—C13—C16—C17	−93.8 (2)
O1—C7—C8—C9	64.77 (16)	C14—C13—C16—C15	−29.05 (16)
C6—C7—C8—C9	−51.83 (17)	C12—C13—C16—C15	86.56 (17)
C7—C8—C9—C11	−137.89 (14)	O6—C15—C16—C17	53.7 (2)
C14—C8—C9—C11	−17.12 (19)	C8—C15—C16—C17	179.54 (17)
C15—C8—C9—C11	94.40 (16)	O6—C15—C16—C13	−126.61 (14)
C7—C8—C9—C10	−11.23 (17)	C8—C15—C16—C13	−0.78 (18)
C14—C8—C9—C10	109.54 (15)	C7—O1—C20—C10	−15.94 (18)
C15—C8—C9—C10	−138.94 (14)	C1—C10—C20—O1	−166.48 (13)
O2—C1—C10—C20	−53.85 (19)	C5—C10—C20—O1	−47.35 (17)
C2—C1—C10—C20	68.20 (17)	C9—C10—C20—O1	67.87 (16)
O2—C1—C10—C5	−174.62 (14)	C6—O3—C21—O7	−12.8 (2)
C2—C1—C10—C5	−52.56 (18)	C6—O3—C21—C22	166.23 (14)
O2—C1—C10—C9	67.26 (18)		

### Hydrogen-bond geometry (Å, °)

**Table:**

*D*—H···*A*	*D*—H	H···*A*	*D*···*A*	*D*—H···*A*
O2—H2O···O5	0.85 (3)	1.75 (3)	2.5539 (19)	156 (2)
O4—H4O···O6^i^	0.82 (3)	2.03 (3)	2.843 (2)	172 (2)
O5—H5O···O2^ii^	0.82 (2)	1.85 (2)	2.6467 (18)	165 (2)
O6—H6O···O3	0.81 (2)	2.07 (3)	2.7743 (18)	145 (2)

Symmetry codes: (i) *x*, *y*−1, *z*; (ii) −*x*+1, *y*+1/2, −*z*.
